# Inflammatory cytokine profiles in erectile dysfunction: a bidirectional Mendelian randomization

**DOI:** 10.3389/fimmu.2024.1342658

**Published:** 2024-04-12

**Authors:** Dongze Liu, Zheng Qin, Bocun Yi, Hongbo Xie, Yunan Liang, Liang Zhu, Kuo Yang, Hongtuan Zhang

**Affiliations:** ^1^ Department of Urology, National Key Specialty of Urology, Second Hospital of Tianjin Medical University, Tianjin Key Institute of Urology, Tianjin Medical University, Tianjin, China; ^2^ Department of Oncology, The Second Hospital of Tianjin Medical University, Tianjin, China

**Keywords:** inflammatory cytokine, erectile dysfunction, bidirectional Mendelian randomization, IP-10, IL-1ra

## Abstract

**Objectives:**

Inflammatory cytokines (ICs) play an important role in erectile dysfunction (ED). Previous studies have demonstrated that most ED patients have high levels of tumor necrosis factor-α (TNF-α), interleukin-6 (IL-6) and interleukin-8 (IL-8). The causality between 41 ICs and ED is investigated using the Mendelian randomization (MR) approach.

**Methods:**

Single nucleotide polymorphisms (SNPs) exposure data of 41 ICs came from a genome-wide association study (GWAS) of 8293 subjects. At the same time, the FINNGEN R9 database provided the ED outcome data containing 2205 ED patients and 164104 controls. MR-Egger (ME), inverse variance weighting (IVW), and weighted median (WM) were applied to conduct the MR study and IVW was taken as the main criterion.

**Results:**

From a genetic perspective, the increase of interferon-inducible protein-10 (IP-10) level significantly increased the risk of ED (P=0.043, odds ratio (OR)=1.269, 95% confidence interval (95%CI): 1.007-1.600), while the increase of interleukin-1 receptor antagonist (IL-1RA) markedly decreased the risk of ED (P=0.037, OR=0.768, 95%CI: 0.600-0.984). Meanwhile, IP-10 (p=0.099) and IL-1RA (p=0.135) failed to demonstrate causality in reverse MR analysis.

**Conclusions:**

Changes in ICs levels will significantly affect the risk of ED, especially IP-10 as a risk component for ED and IL-1RA as a protective component for ED. In the future, we can achieve targeted treatment and prevention of ED by intervening with specific inflammatory factors.

## 1 Introduction

Erectile dysfunction (ED) is generally referred to as a failure to produce or satisfy sexual function needs for an erection lasting more than 3 months (1). ED is currently recognized as a major health issue for men worldwide, often having adverse effects on patients’ quality of life (QoL). Globally, it is projected that there will be 322 million ED cases by 2025 (2, 3).

The essence of ED is usually regarded as a vascular disease, in which vasculitis plays an important role. Previous studies have observed that ED patients exhibit an increase in their inflammatory marker levels, including interleukin-6 (IL-6) and von Willebrand Factor (vWF). Oral administration of the anti-ED drug tadalafil can also lead to dramatic changes in the levels of inflammatory factors (4). Another study analyzed the full-gene mRNA pathway enrichment analysis of vascular matrix components in patients with ED and found that the upregulated differential genes in ED were enriched in the inflammatory response, immune regulation, and activation of interleukin 7 and complement (5). The above studies revealed the special status of inflammatory factors in ED patients. At the same time, the above-mentioned studies also suggested that early detection and treatment of ED may depend on the equilibrium of pro- and anti-inflammatory molecules (4). However, changes in the amount of pro- and anti-inflammatory factors in ED patients are complex. One study showed that the amount of tumor necrosis factor-α (TNF-α) in ED patients was increased, while interleukin-10 (IL-10) expression levels were decreased (6). Although another study observed interleukin-8 (IL-8) and interleukin-18 (IL-18) levels in cavernous blood and venous blood differed, there was no difference in the predictability of clinical erection scores (7). Therefore, the risk role of each specific circulating inflammatory factor in ED needs to be further determined. Importantly, inflammatory cytokines (ICs) are affected by confounding factors such as metabolism and cardiovascular status, which further increases the complexity of ICs research (8). Therefore, the specific cause-and-effect relationship between ICs and ED has not been established. It is necessary to systematically elucidate the direct relationship between 41 ICs and ED to further explore pathogenic mechanisms and specific therapeutic targets for ED.

In mendelian randomization (MR), single nucleotide polymorphisms (SNPs) can be viewed as instrumental variables (IVs) in genetics to perform causal analysis (9). IVs can be viewed as a useful tool for evaluating the causality between exposure and outcome by efficiently avoiding the effects of confounding factors in previous epidemiological work (10). In our survey, our team used the data of 41 ICs in the GWAS database and the ED data in the FINNGEN database to reveal the exact role of ICs in ED through bidirectional MR analysis.

## 2 Methods

### 2.1 Research design

**Figure 1:**
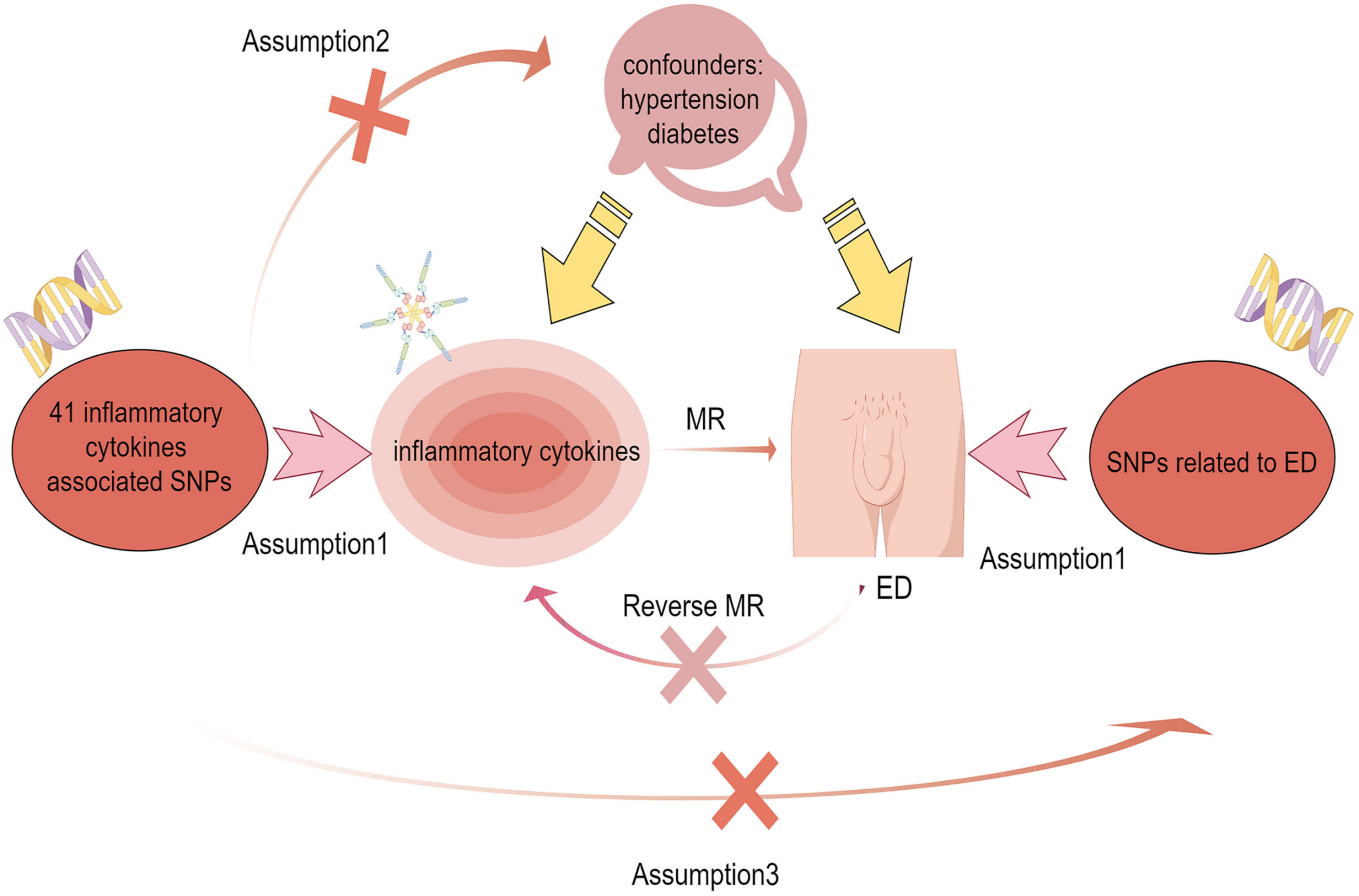
Flow chart of bidirectional mendelian randomization between ICs and ED. Assumption 1: There is a strong correlation between IVs and ICs. Assumption 2: IVs are independent of confounders including hypertension and diabetes to influence ED. Assumption 3: IVs only influence the ED through the ICs.

SNPs as IVs in genetic variation were used in MR analysis to analyze causality between 41 ICs and ED (11). MR follows 3 basic principles: (1) A Strong correlation has to exist among IVs and exposures. (2) IVs are not subject to confounding factors. (3) IVs influence on results by exposure only (12). The workflow diagram is shown in **Figure 1**.

### 2.2 Data acquisition and selection of IVs

41 ICs-related SNPs were derived from a published GWAS study containing the Young Finns Cardiovascular Risk Study (YFS) and the FINRISK research (FINRISK 1997/FINRISK 2002) (13, 14). ED-related data came from the FINNGEN R9 database (https://www.finngen.fi/en/). The data were open and accessible without requiring additional approval. First, we chose p< 5*10-8 as the relevance filter cutoff point for SNPs associated with inflammatory cytokines. Because very few SNPs were found when ICs were employed as the exposure, a generous threshold (p< 5*10-6) was adopted (15). Second, we have eliminated the disequilibrium of SNPs linkage to ensure their independence (r^2^<0.001 and kb=10000) (16). The final step was to calculate F statistics and all values were greater than 10 to eliminate weak bias caused by IVs (17). Then we accessed the data resources on the Phenoscanner website to eliminate confounding SNPs associated with hypertension and diabetes (18). To obtain enough SNPs as IVs for reverse MR analysis, we chose p< 5*10^-5^ as the relevant filter cutoff point for SNPs associated with ED and set the linkage imbalance parameters as follows (r^2^<0.01 and kb=5000).

### 2.3 Statistical study

Weighted median (WM), inverse variance weighting (IVW), and MR-Egger (ME) methods are employed to evaluate the causality between 41 ICs and ED. The main approach with the highest statistical power is IVW which considers all genetic variants to be valid IVs (19). Although the statistical power of ME and WM is weaker than that of IVW, they have a higher tolerance for invalid IVs (20). At the same time, during the evaluation of causal effects by the ME method, the regression intercept of ME can also be used as a basis for testing horizontal pleiotropy (21). MRPRESSO was also used to identify horizontal pleiotropy and improve potential pleiotropy via the removal of outliers (22). The heterogeneity of IVs can be detected by using the Cochrane Q statistic (23). We assessed the overall stability with a leave-one-out approach to our research findings. When the IVW showed statistical significance (P <0.05), despite the ME and WM methods showing no statistical significance, it is still seen as favorable if the β were consistently in the same direction (24). The ‘forestploter’ package was used to draw forest plots, the ‘circlize’ package and the ‘ComplexHeatmap’ package were used to draw circular heat maps, the ‘TwoSampleMR’ package and MRPRESSO package were used to perform MR analysis and display the results of P value. The R platform was used for all MR analyses (4.2.3).

## 3 Results

### 3.1 Causality between 41ICS and ED

**Figure 2:**
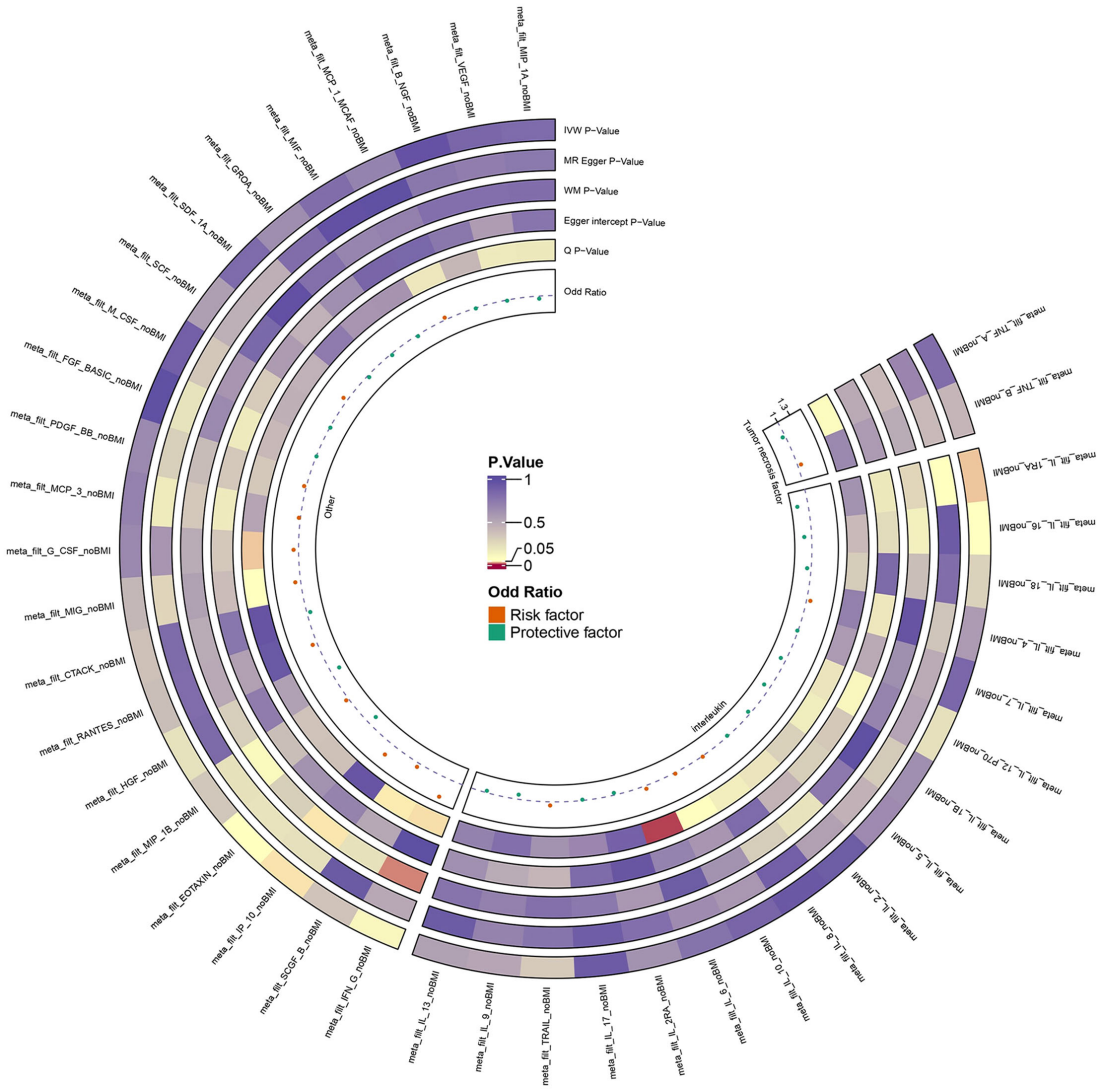
Ring heat map of 41 ICs and ED.

We extracted 41 ICs exposure data and ED outcome data and successfully eliminated linkage disequilibrium and palindrome sequences. Through the harmonize function, SNPs which were shared by 41 ICs and ED were obtained to explore causality. The results of our analysis revealed that among the 41 ICs, only IP-10 and IL-1RA had significant p-values in the IVW results (**Supplementary Material S1**). We presented the analysis results of 41 ICs in the form of an annular heat map (**Figure 2**). IP-10 and IL-1RA contained 9 and 8 SNPs respectively. We checked the above SNPs through the Phenoscanner website and found that they were not associated with common interfering factors of ED, including hypertension and diabetes (25, 26). All SNPs had F values ranging from 20.9 to 32.2, indicating that IVs were valid.

### 3.2 Causality between IP-10, IL1-RA and ED

**Figure 3:**
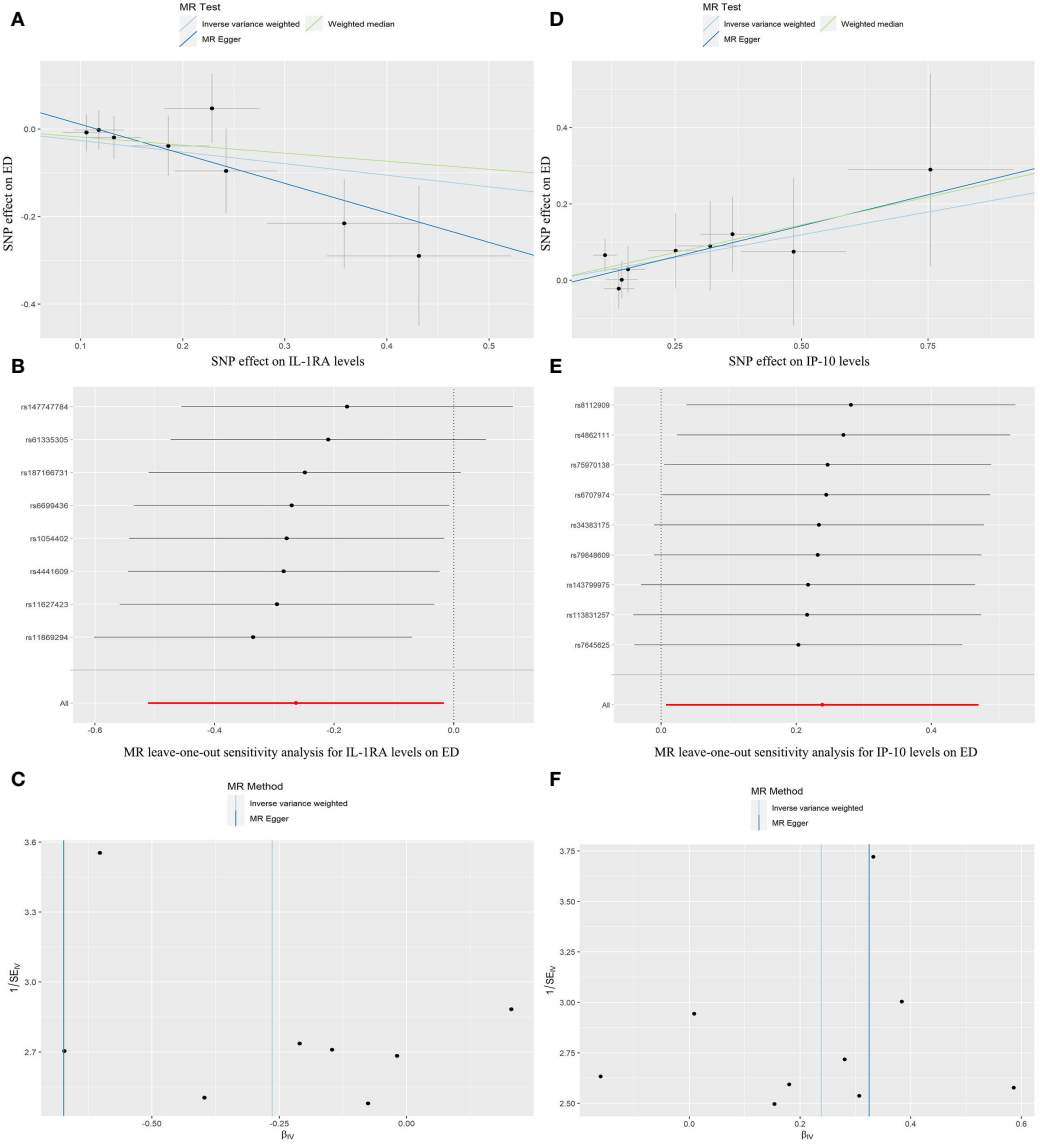
Scatter plots, forest plots, and funnel plots represent the corresponding risk relationships between SNPs of IL-1RA **(A–C)**, IP-10 **(D–F)**, and ED.

**Figure 4:**
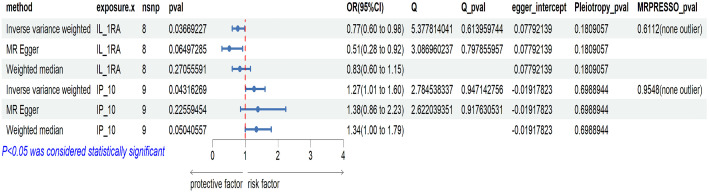
Forest plot of MR analysis results between IL-1RA, IP-10, and ED.

**Figure 3** illustrates the causality between genetically predicted ICs and ED. The results indicated that high levels of circulating IL-1RA in the IVW approach will result in a significant decrease in the likelihood of experiencing ED (p = 0.037, odds ratio (OR) = 0.768, 95% confidence interval (95%CI) = 0.600–0.984). According to Cochran’s Q test, there was no evidence of heterogeneity in subsequent sensitivity analyses (p = 0.614). Similarly, neither ME nor MRPRESSO presented horizontal pleiotropy (ME-intercept = 0.078, ME-intercept P = 0.181; MRPRESSO test P = 0.6112). MRPRESSO analysis also showed that there were no outliers in IL-1RA related SNPs. At the same time, the high level of circulating IP-10 in the IVW method will result in a markedly raised risk of ED (p = 0.043, OR = 1.269, 95% CI = 1.007–1.600). Heterogeneity was not detected according to Cochran’s Q test (p = 0.947). Horizontal pleiotropy was also not presented in ME or MRPRESSO (ME intercept = -0.019, ME intercept P = 0.699; MRPRESSO test P = 0.955). There were no SNPs outliers in IP-10 in MRPRESSO analysis. The above analysis results were displayed in the form of forest plots (**Figure 4**).

### 3.3 Reverse MR between IP-10, IL-1RA and ED

To exclude the suspicions of reverse causation, we used the SNPs of ED as exposure data and the IP-10 and IL-1RA related SNPs as outcome data, respectively. The findings of the reverse MR study suggested that there was no causality between IP-10 (p=0.099), IL-1RA (p=0.135), and ED in reverse MR analysis (**Table 1**). Therefore, our conclusion did not suggest reverse causation.

**Table 1:** Reverse MR analysis results between IP-10, IL-1RA, and ED.

ICs	method	nsnp	b	se	pval
IP-10	MR Egger	4	-0.03852	0.19589	0.862287
IP-10	Weighted median	4	0.088535	0.121142	0.464878
IP-10	Inverse variance weighted	4	0.145493	0.08821	0.099066
IP-10	Simple mode	4	0.078284	0.173298	0.682122
IP-10	Weighted mode	4	0.046072	0.148221	0.776274
IL-1RA	MR Egger	5	-0.16618	0.180248	0.42455
IL-1RA	Weighted median	5	0.120002	0.113737	0.291384
IL-1RA	Inverse variance weighted	5	0.13485	0.090311	0.135391
IL-1RA	Simple mode	5	0.27039	0.175856	0.198974
IL-1RA	Weighted mode	5	-0.0325	0.181223	0.866399

## 4 Discussion

ED is a serious health topic faced by men worldwide. The prevalence rates of middle-aged and elderly people in the United States, Europe, and Asia are 52%, 30%, and 63%, respectively. Importantly, ED patients have a significant psychological burden, with anxiety and depression rates of 79.82% and 79.56%, respectively (27). However, little has been identified about the pathogenic mechanisms and causes of ED. Observational studies have drawbacks, such as confounding factors, measurement error, and reverse causation until now. Randomized controlled trials also suffer from the interference that risk factors cannot be assigned randomly. Mendelian randomization uses measurable genetic variation as IVs to demonstrate causality thus avoiding the above concerns and making the results more convincing. In our study, we used genetic data from a public database to conduct a two-sample MR assessment to confirm whether the levels of 41 ICs were related to ED risk. Our results revealed that there is a causality between high circulating quantities of IP-10 and an increased risk of ED, whereas massively expressed IL-1RA is strongly associated with a reduced risk of ED.

IP-10 is a chemotactic cytokine involved in inflammation and immune responses. It serves as a chemoattractant for multiple cell types including dendritic cells, macrophages, monocytes, and T cells. Endothelial cells, monocytes, and fibroblasts secrete IP-10 upon receiving the upstream signal interferon-γ (IFNγ). In addition, IP-10 can engage with the cell surface chemokine receptor 3 (CXCR3) to inhibit the formation of blood vessels and bone marrow colonies to achieve the ultimate anti-tumor effect (28, 29). At present, there are few basic research and clinical observational studies to reveal the relationship between IP-10 and ED, and only one study has observed a transient elevation of IP-10 expression initially and a sharp decline later in the treatment of ED mice with vascular endothelial growth factor (VEGF), suggesting that IP-10 could be a risk component and prognostic marker in ED (30). Through MR we found that high-level circulating IP-10 led to an increased risk of ED.

Interleukin-1 beta (IL-1β) and interleukin-1 alpha (IL-1α) have potent pro-inflammatory action in infections and autoimmune diseases. IL-1RA binds to the IL-1 receptor competitively to suppress the activity of IL-1α and IL-1β (31). Therefore, IL-1RA protein is considered a marker and determinant of IL-1 induced inflammatory response (32, 33). When there is an out of balance between IL-1 and IL-1RA, tissue reactivity will be enhanced, resulting in abnormalities in tissue structure and function (34, 35). It is found that IL-1RA involves the advancement of numerous human diseases, including inflammatory bowel disease, arthritis, lung disease, liver disease, tumors, diabetes, and arterial disease. IL-1RA acts as a protective factor in the above diseases and greatly improves the prognosis of patients. Therefore, IL-1RA has been used as a new therapeutic target (36). At present, the role and mechanism of IL-1RA in ED have not been revealed. IL-1 has been reported to cause endothelial dysfunction and even mediate systemic inflammation and venous thromboembolism (37). Vascular ED is considered an early manifestation of vascular endothelial injury (38). Our MR analysis preliminarily drew a potential association between IL-1RA and ED: high circulating levels of IL-1RA would help reduce the risk of ED. Therefore, IL-1RA is expected to serve as a potential treatment for ED by blocking the inflammatory damage of IL-1.

Our study exhibits the subsequent strengths. Our team conducted the MR analysis between ICs and ED for the first time through genetic instrumental variables, and the data were all derived from public databases, which provided a strong guarantee for this study. Importantly, this study will help determine the importance of ICs in the etiology of ED. Furthermore, the IVs we studied were independent of common confounding factors to provide reliable causal relationships. At the same time, our results were also shown to be robust through a series of sensitivity analyses. Finally, we adopted a looser threshold of 5*10^-6^ than 5*10^-8^ to enhance the feasibility. Meanwhile, our study still has some limitations. First, MR research uses genetic data to demonstrate causal relationships and cannot rule out the impact of non-genetic factors on ED, such as living environment and lifestyle habits. Second, even if the F statistics of IVs exceed 10, the presence of IVs with a genome-wide threshold P < 5 × 10^-6^ can still potentially introduce a subtle instrumental variable bias. Third, the information in this research is exclusively from the European population, and the research result cannot be generalized to all populations. Other populations still need to be observed. Finally, our study only initially revealed the causality between ICs and ED according to MR analysis. In the future, we need further *in vivo* and *in vitro* experiments to verify our conclusions and explore possible mechanisms.

## 5 Conclusions

This study establishes a link between the risk of ED and the circulating volume of IP-10 and IL-1RA. Understanding the changes in the levels of the ICs is crucial for evaluating the prognosis of patients with ED. In addition, the above findings will further help us understand the pathogenesis of ED and thus improve our clinical treatment strategies. In particular, IP-10 and IL-1RA have the potential to become a new therapeutic target for ED.

## Data availability statement

The datasets presented in this study can be found in online repositories. The names of the repository/repositories and accession number(s) can be found in the article/**Supplementary Material**.

## Author contributions

DL: Conceptualization, Formal analysis, Methodology, Writing – original draft. ZQ: Methodology, Writing – original draft. BY: Data curation, Formal Analysis, Methodology, Software, Writing – review & editing. HX: Data curation, Formal Analysis, Methodology, Writing – review & editing. YL: Data curation, Formal Analysis, Methodology, Writing – review & editing. LZ: Writing – review & editing. KY: Writing – review & editing. HZ: Funding acquisition, Project administration, Resources, Writing – review & editing.
